# An exploration of the most frequent comorbidities in patients with mobile phone addiction

**DOI:** 10.1192/j.eurpsy.2024.857

**Published:** 2024-08-27

**Authors:** O. Vasiliu, D. G. Vasiliu

**Affiliations:** ^1^Psychiatry, Dr. Carol Davila University Emergency Central Military Hospital; ^2^Regina Maria Foundation, Bucharest, Romania

## Abstract

**Introduction:**

Mobile phone addiction (MPA) has been associated in the literature with various psychiatric comorbidities and psychological risk factors, which indicates the need to screen these patients for multiple disorders. However, a clear protocol for the evaluation of individuals with an MPA does not yet exist, therefore, investigating the most prominent risks for comorbidities is considered necessary from the perspective of developing structured methods of assessment.

**Objectives:**

The main objective of this review was to determine the available existence able to describe the most common comorbidities in individuals presenting with MPA.

**Methods:**

Data regarding MPA were collected from the main medical electronic databases (PubMed, Cochrane, Clarivate/Web of Science), but also from other sources (main engines research and grey literature). All published papers between January 2000 and July 2023 were included in the primary selection, if they corresponded to the paradigm „mobile phone addiction”/”cell phone addiction”/”mobile phone dependence” and „comorbidity”/”dual diagnosis”.

**Results:**

Based on the review of six papers, the most frequently reported comorbidity in MPA patients were substance use disorders (mainly nicotine and cannabis) and other behavioral addictions (especially problematic Internet use). Other symptoms or syndromes reported in the literature as co-occurring with MPA were anxiety, depression, high levels of stress-related pathology, sleep disturbances, emotional instability, and somatization. Overall lower levels of mental health were reported in patients with MPA. A heterogeneity in the results of these epidemiological studies was observed because of the different instruments administered and the populations explored.

**Conclusions:**

The screening for detection of comorbid disorders or psychological problems in patients with MPA is important because the case manager should integrate all this information into a therapeutic strategy.

**Disclosure of Interest:**

None Declared

